# Ubiquitin-specific protease 22 is critical to in vivo angiogenesis, growth and metastasis of non-small cell lung cancer

**DOI:** 10.1186/s12964-019-0480-x

**Published:** 2019-12-16

**Authors:** Keqiang Zhang, Lu Yang, Jinhui Wang, Ting Sun, Yuming Guo, Rebecca Nelson, Tommy R. Tong, Rajendra Pangeni, Ravi Salgia, Dan J. Raz

**Affiliations:** 1 Division of Thoracic Surgery, City of Hope National Medical Center, Duarte, California USA; 2 Department of System Biology, City of Hope National Medical Center, Duarte, California USA; 3 The Integrative Genomics Core Laboratory of Department of Molecular Medicine, City of Hope National Medical Center, Duarte, California USA; 4 Department of Surgery, the General Hospital of Ningxia Medical University, Yinchuan, China; 5 Division of Comparative Medicine, City of Hope National Medical Center, Duarte, CA USA; 6 Department of Pathology, City of Hope National Medical Center, Duarte, California USA; 7 Division of Biostatistics, City of Hope National Medical Center, Duarte, California USA; 8 Department of Medical Oncology & Therapeutics Research, City of Hope National Medical Center, Duarte, California USA

**Keywords:** Non-small cell lung cancer (NSCLC), USP22, Angiogenesis, Epithelial-mesenchymal transition (EMT), Growth, Metastasis, Therapeutic target

## Abstract

**Background:**

Loss of monoubiquitination of histone H2B (H2Bub1) was found to be associated with poor differentiation, cancer stemness, and enhanced malignancy of non-small cell lung cancer (NSCLC). Herein, we investigated the biological significance and therapeutic implications of ubiquitin-specific protease 22 (USP22), an H2Bub1 deubiquitinase, in non-small cell lung cancer (NSCLC).

**Methods:**

USP22 expression and its clinical relevance were assessed in NSCLC patients. The effects of USP22 knockout on sensitivity to cisplatin and irradiation, and growth, metastasis of NSCLC xenografts, and survival of cancer-bearing mice were investigated. The underlying mechanisms of targeting USP22 were explored.

**Results:**

Overexpression of USP22 was observed in 49.0% (99/202) of NSCLC tissues; higher USP22 immunostaining was found to be associated with enhanced angiogenesis and recurrence of NSCLC. Notably, USP22 knockout dramatically suppressed in vitro proliferation, colony formation; and angiogenesis, growth**,** metastasis of A549 and H1299 in mouse xenograft model, and significantly prolonged survival of metastatic cancer-bearing mice. Furthermore, USP22 knockout significantly impaired non-homologous DNA damage repair capacity, enhanced cisplatin and irradiation-induced apoptosis in these cells. In terms of underlying mechanisms, RNA sequencing and gene ontology enrichment analysis demonstrated that USP22 knockout significantly suppressed angiogenesis, proliferation, EMT, RAS, c-Myc pathways, concurrently enhanced oxidative phosphorylation and tight junction pathways in A549 and H1299 NSCLC cells. Immunoblot analysis confirmed that USP22 knockout upregulated E-cadherin, p16; reduced ALDH1A3, Cyclin E1, c-Myc, and attenuated activation of AKT and ERK pathways in these cells.

**Conclusions:**

Our findings suggest USP22 plays critical roles in the malignancy and progression of NSCLC and provide rationales for targeting USP22, which induces broad anti-cancer activities, as a novel therapeutic strategy for NSCLC patient.

## Background

Lung cancer is the leading cause of cancer-related death worldwide [1]. Non-small-cell lung cancers (NSCLCs), the most common lung cancers, are defined as a group of distinct disease with genetic and cellular heterogeneity. The two predominant NSCLC histological phenotypes are adenocarcinoma (~ 50%) and squamous cell carcinoma (~ 40%) [1]. KRAS and epidermal growth factor receptor (EGFR) mutations are the most frequent oncogenic drivers discovered in lung adenocarcinomas. For lung squamous cell carcinoma, genes such as fibroblast growth factor receptor 1 to 3 (FGFR1–3)**,** and genes in the phosphoinositide 3-kinase (PI3K) pathway seem to be more commonly mutated in lung squamous carcinoma [2, 3]. Many targeted therapies against kinases EGFR, anaplastic lymphoma kinase (ALK), and c-ros oncogene 1 receptor tyrosine kinase (ROS1) have been developed with compelling clinical proofs of concept and various survival benefits; however, treatment responses are typically short-lived [2, 3]. To date, no efforts at targeting KRAS have been proven to be successful. Therefore, despite advances in targeted therapies and immunotherapy, most lung cancers are still incurable and new therapies are needed [4]. Notably, recent studies have demonstrated that epigenetic modifications work with genetic mechanisms and play critical roles in carcinogenesis and progression and treatment resistance of cancers with different genetic backgrounds and oncogenic drivers, which makes them attractive and novel therapeutic candidate targets for cancers [5, 6]. Ubiquitin-specific peptidase 22 (USP22), a subunit of the human SAGA (Spt-Ada-Gcn5-Acetyltransferase) complex, is an ubiquitin hydrolase, catalyzing the removal of the mono-ubiquitin moiety from histone H2B (H2Bub1) [6, 7]. Interestingly, an earlier study identified that USP22 is one of an 11-gene transcriptional signature which was associated with aggressive growth, metastasis, and therapy resistance in a number of human cancers including lung cancer [8]. Frequent overexpression of USP22 protein was further discovered in various aggressive cancers including breast and colon cancers, and was demonstrated to be associated with poor prognosis of cancer patients [9, 10]. USP22 is required for activated transcription and cell-cycle progression, and critical for cell proliferation [7]. We recently showed that frequent loss of H2Bub1 is significantly associated with aggressive tumor biology in lung adenocarcinoma [11], and USP22 protein is enriched and plays critical role in cancer stem cells isolated from primary lung adenocarcinoma [12]. USP22 has been proposed as a putative cancer stem cell marker and a novel drug target in cancers including lung cancer [13]. However, studies to date also suggest USP22 may function as a tumor suppressor in cancers. A study by digging The Cancer Genome Atlas (TCGA) data found that USP22 gene is much more frequently lost (homozygous or heterozygous loss) than gained in many cancer types [14, 15]. Especially, a recent study demonstrated that USP22 deficiency leads to myeloid leukemia upon oncogenic KRAS activation through a PU.1 dependent mechanism [16]; and data of another recent study also showed that USP22 loss promotes colorectal cancer by elevating mTOR activity, indicating USP22 may function as a tumor suppressor in colorectal cancer [17]. These data suggest that there may be multiple roles of USP22 in initiation and development of various cancers that remain to be fully elucidated.

Although overexpression of USP22 has been observed in various cancers including lung cancer, our understanding of potential roles of USP22 in NSCLC is still largely incomplete. In this study, we assessed the status, biological significance and therapeutic implications of USP22, and explored the mechanism by which USP22 may affect cancer-associated signaling pathways in NSCLC cells. Our findings reveal that USP22 plays a critical oncogenic role and represents a potential therapeutic target in NSCLC, and targeting USP22 will bring broad antitumor effects through suppression on multiple signaling pathways associated with cancer progression, which warrants further study.

## Materials and methods

### Patients selection and clinical data collection

This study was reviewed and approved by the Institutional Review Board (IRB) of City of Hope National Medical Center. A cohort of 240 NSCLC patients who underwent surgical resection for curative intent between 2002 and 2014 without preoperative chemotherapy or radiation therapy were included, and patients’ clinical characteristics are summarized in Table 1. Tissue microarrays were created using cancer and matched normal tissues.

**Table 1:** The correlation of USP22 immunostaining with the clinical features of NSCLC patients

Variable*		Negative N (%)	1 + N (%)	2 + N (%)	3 + N (%)	*P*-value
Age Group	<= 60 year	11 (17)	3 (10)	12 (21)	11 (25)	0.4045
	> 60 year	54 (83)	27 (90)	46 (79)	33 (75)	
Sex	Male	32 (49)	17 (56)	27 (47)	24 (56)	0.7232
	Female	33 (51)	13 (44)	31 (53)	19 (44)	
Tobacco History	Never used	8 (14)	9 (32)	6 (13)	3 (9)	0.0445
	Previous use	34 (58)	16 (57)	31 (65)	17 (50)	
	Current use	17 (29)	3 (11)	11 (23)	14 (41)	
Histology	Adenocarcinoma	40 (62)	18 (60)	34 (59)	24 (56)	0.9469
	SCC	25 (38)	12 (40)	24 (41)	19 (44)	
Grade	Well Differentiated	5 (8)	4 (14)	9 (16)	8 (19)	0.5089
	Moderately Differentiated	28 (44)	15 (53)	27 (48)	16 (38)	
	Poorly Differentiated	30 (48)	9 (33)	20 (36)	18 (45)	
Path Stage	I	39 (61)	17 (56)	33 (59)	34 (79)	0.0883
	II	16 (25)	7 (24)	8 (14)	3 (7)	
	III	9 (14)	6 (20)	15 (27)	6 (14)	
Recurrence	No	51 (80)	26 (87)	51 (91)	30 (70)	0.0439
	Yes	13 (20)	4 (13)	5 (9)	13 (30)	

*Patients who were never disease free were excluded from the analysis (*n* = 9)

### Immunohistochemistry analysis

Tissue arrays include about 240 tumor samples each including two spots and their matched noncancerous tissues were used for immunohistochemistry (IHC) analysis. Monoclonal anti-USP22 antibody (ab195289) was from Abcam (Cambridge, MA) and monoclonal ati-H2Bub1 (MABE453) was from EMD Millipore (Burlington, MA). The mouse monoclonal antibodies against E-Cadherin (4A2), Vimentin (D21H3), Sirt1 (1F3), p16 INK4A (D7C1M), p53 (7F5), ALDH1A3 (ab12915), total and cleaved poly (ADP-ribose) polymerase (PARP) (Asp214, 19F4), Cyclin D1 (92G2), trimethylated H3K4 (C42D8) and H3K79 (Cat#: 4260) were purchased from Cell Signaling Technology (Beverly, CA USA) and Abcam. IHC was performed as described previously [11, 18]. USP22 IHC staining was graded as negative (0), if < 1% cells displayed positive nuclear staining. Those cases with > 1% of tumor cells showing nuclear staining for USP22 were classified as positive, and graded as 1+ (1–5%), 2+ (5–24%), and 3+ (> 25% of the cells stained positive) as described previously [11, 18]. Microvessel density (MVD) in lung cancer tissues was evaluated after immunostaining endothelial cells with antibody against human CD31 (The JC70 Mab from DAKO), and ranked as rare (0), low (1+), medium (2+), and high (3+) for MVD as described previously [19].

### Cell culture, proliferation, migration and matrigel invasion, and apoptosis assays

Human lung cancer cell lines: A549 (KRAS/G12S, p53 wild-type), H1299 (NRAS/Q61K, p53-null) cells were cultured in regular DMEM or RPMI medium. For proliferation assessment, cells were seeded in 96-well plates in 5 replicates at densities of 3.0 × 10^3^ cells per well, and were measured at 72 h using cell counting kit-8. Apoptosis was measured by flow cytometry analysis of Alexa Fluor 488-labeled Annexin-V and propidium iodide staining, according to the manufactory’s protocol. Migration and matrigel invasion assays were performed as described previously [20].

### siRNA transfection and RNA-seq data analysis

A USP22 siRNAs: Cat No.10620318 (5′-GGCAUCUCAGGAGGAUGCCAAUGAA-3′) purchased from Thermo Fisher Scientific Corporation (Carlsbad, CA) were applied to transiently silence USP22 expression using the protocol we described previously [11, 18]. To evaluate the biology of USP22 in NSCLC, we generated two individual colonies of homologous USP22-knockout (USP22−/−) A549 and H1299 cells by CRISPR/Cas9 system. Lentivectors expressing CRISPR/Cas9 single guide RNA (sgRNA) 1 and 2 (targeting sequence 124-TACCAGTGCTTCGTGTGGAG, and 272-ACGAGCATGCGAAGGCGAAG) were purchased from Applied Biological Materials Inc. (Richmond, BC, Canada). A549 and H1299 cells were first infected with each lentivirus expressing sgRNA and then transfected with plasmid expressing Cas9 nuclease, and then were finally selected with 0.5–1 μg/mL puromycin to generate USP22−/− A549 and H1299 cell clones by sgRNA1 and sgRNA2 respectively, named as USP22−/−C1 and -C2 [12]. Transcriptome libraries and RNA sequencing analysis were performed according to the Illumina Genome Analyzer II (Illumina, San Diego, CA, USA) manufacturer’s instruction with minor modifications as we described previously [11, 18].

### DNA repair assays

Reporter cell lines for green fluorescent protein (GFP)-based DNA damage repair assays were established by first transfection of the parent and USP22−/− H1299 cells with the pimEJ5GFP reporter plasmid for non-homologous end joining (NHEJ) or the pHPRT-DRGFP reporter plasmid for homologous repair (HR), and following transfection with a predetermined mixture of pCBA-Scel plasmid to express I-Scel endonuclease to make DNA double-strand breaks (DSBs) in the reporter plasmids and a plasmid to express tdTomato protein that served as the control for transfection efficiency, after culturing for another 48 h, 5 × 10^5^ cells per transfection were analyzed by fluorescence-activated cell sorting (FACS) to measure GFP and tdTomato protein-positive cells, respectively [21]. The DNA damage repair activities were calculated and presented as the ratio of GFP-positive to tdTomato-positive cells among whole cells as described previously [21].

### Cisplatin and irradiation treatment

Cells were seeded at a density of 2 × 10^5^ cells/well in 6-well plates containing RPMI, supplemented with 10% FBS. After overnight incubation, cells were treated for 72 h with 5 μM Cisplatin, and cells were collected for apoptosis analysis by FACS. For irradiation, cells were irradiated (0–10 Grays), as monolayer, using the Shepherd Mark 168 Irradiator (JL Shepherd, San Fernando, CA, USA) dose rate of 70.6 rad/min at room temperature, and maintained at 37 °C in the incubator with 5% CO_2_ for 2 h, 24 h to extract protein for Western blot analysis, or for 72 h to collect cell for apoptosis analysis.

### In vivo growth and experimental lung, liver metastasis models

Subcutaneous xenografts of cancer cells in NOD/SCID/IL2Rgamma null mice (NSG, 24–27 g, 8–10 weeks of age, 8 mice/group) from Jackson Labs (Bar Harbor, ME) were generated with 5 × 10^6^ cells and measured as previously described [18]. For metastatic cancer model, the parent and USP22−/− cancer cells were injected into the tail veins of 10 mice respectively. About 5 weeks after injection, mice were euthanized by CO_2_ inhalation, and their lungs and livers were excised. Tumor nodules formed in lung and liver were quantified, and tumor weigh were measured. For survival studies, two groups of 10 NSG mice were injected with the parent cells or USP22−/− cancer cells. For in vivo xenograft experiments, half of 8–10 mice were injected with USP22−/− C1, and another half were injected with USP22−/− C2. On day 35, surviving mice were euthanized and lungs and livers were excised and fixed in formalin solution for 24 h at room temperature and examined for lung and liver metastasis as described above. All mouse studies were performed in the animal facility at City of Hope accordance with institutional guidelines.

### Statistical analysis

All experiments were performed in duplicates or triplicates and repeated at least two times in each experiment. Two group comparisons were analyzed for variation and significance using a Student’s *t-*test or Pearson χ^2^ test. All data shown are mean ± standard deviation (SD). Kaplan-Meier analysis was used to compare overall survival of metastatic cancer-bearing mice and patients in each subgroup. Correlation between USP22 IHC and MVD/CD31 was analyzed by Spearman’s rank correlation analysis. Statistical significance was set at *P <* 0.05.

## Results

### Upregulation of USP22 is associated with cancer recurrence in NSCLC

**Fig. 1:**
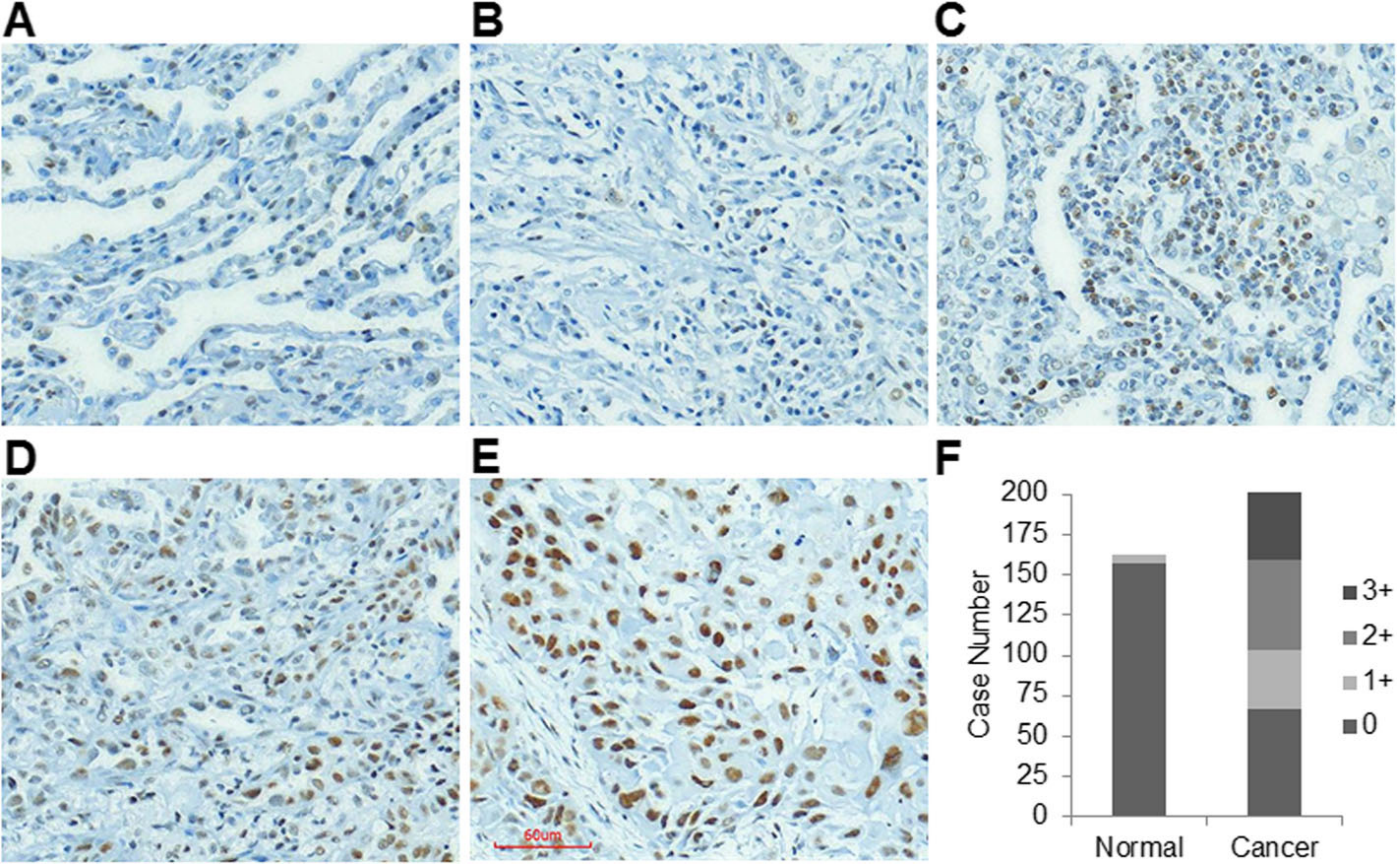
Upregulation of USP22 in NSCLC tissues. **a**. USP22 IHC staining in normal lung tissue, no nuclear USP22 staining is found in the normal lung tissues. Photomicrographs of four representative NSCLC sections stained for **b**. 0, **c**. 1+, **d**. 2+, **e**. 3+ USP22 nuclear immunostaining (magnification, × 200). **f**. Histogram of the case numbers of normal and NSCLC tissues in which USP22 was scored as 0 to 3+

To explore the significance of USP22 expression in NSCLC tissues, we first examined USP22 protein in 202 cancer tissues and their matched noncancerous lung tissues by immunohistochemical analysis. IHC analysis shows USP22 nuclear immunostaining was undetectable in the vast majority of normal lung tissues (Fig. 1a), and scant, weak nuclear USP22 immunostaining was observed in a very small part of normal tissues (6/163, 163 cancer tissues had the paired normal tissues), and a moderate to strong nuclear immunostaining of USP22 were found NSCLC tissues. IHC analysis showed that USP22 was undetectable (scored as 0, Fig. 1b) in 33.2% (67/202), while USP22 levels in 17.8% (36/202), 27.7% (56/202), and 21.3% (43/202) of cancer cases were scored as 1+ (Fig. 1c), 2+ (Fig. 1d), 3+ (Fig. 1e) respectively. Figure 1f summarizes the case numbers of tissues with different USP22 nuclear immunostainings of both matched non-cancerous and cancer tissues. Statistical analysis showed that the intensities of USP22 immunostainings were positively associated with cancer recurrence (*P* = 0.044) and a trend toward advanced stage (*P* = 0.088) (Table 1) in these NSCLC tissues.

### Downstream targets and signaling pathways of USP22 in NSCLC

**Fig. 2:**
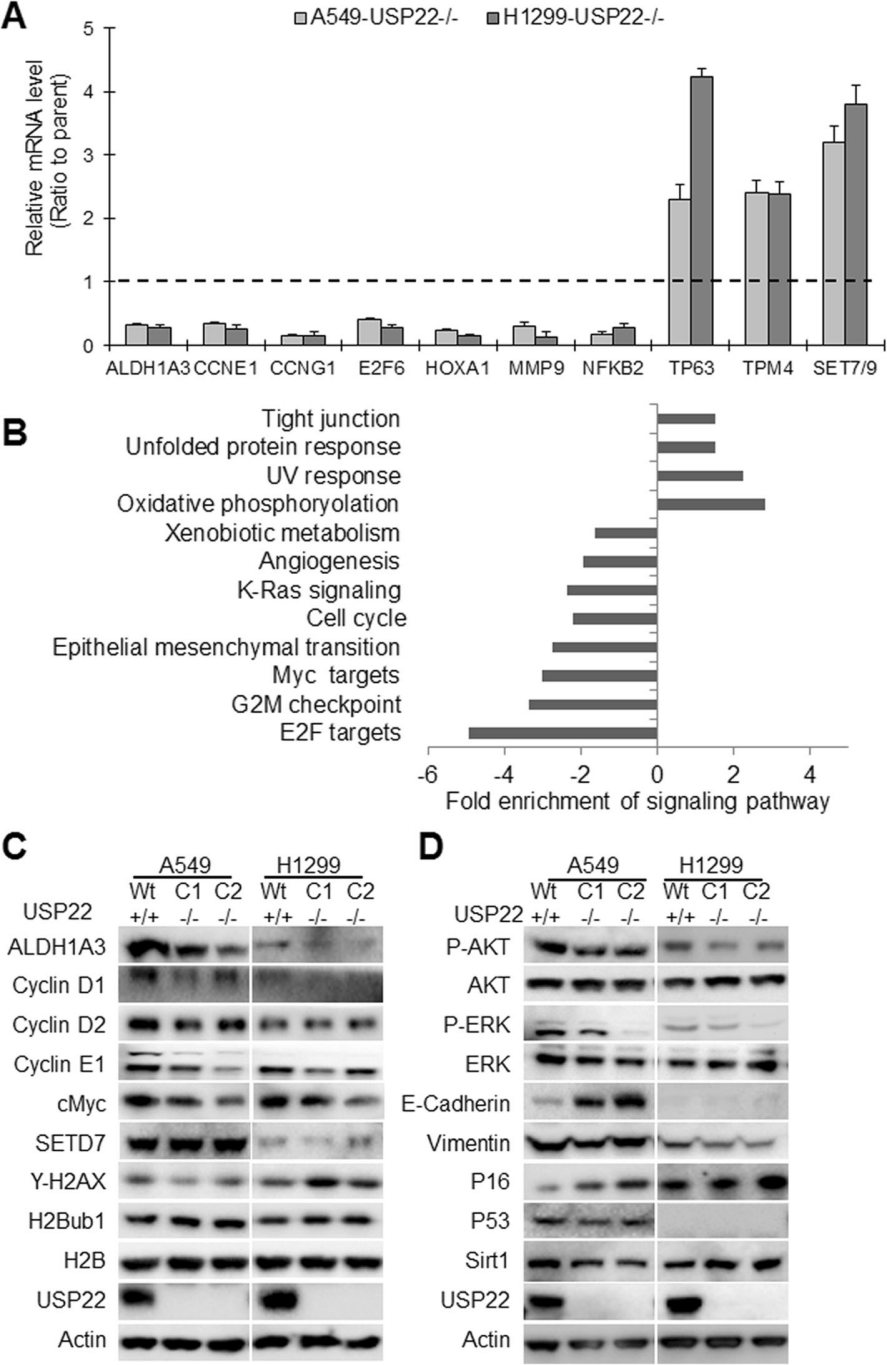
Alternated gene expression and signaling pathways in A549 and H1299 cells upon USP22 knockout. **a**. qRT–PCR analysis of selected differentially expressed genes in USP22−/− A549 and USP22−/− H1299 cancer cells compared to the parent cells. The level of each gene in USP22−/− cells is the average ratio of triplicate samples, and is presented as the ratio to the parent cancer cells (USP22+/+); *P* < 0.05, compared with the parent cells. **b**. Selected signaling pathways that were enriched in USP22−/− A549 and USP22−/− H1299 cancer cells (*P* < 0.05, FDR < 5%). **c**. Western blot analysis of differentially expressed ALDH1A3, Cyclin D1, Cyclin D2, Cyclin E2, c-Myc, and SETD7 in USP22−/− cancer cells. **c**. Western blot analysis of AKT, ERK, E-Cadherin, Vimentin, p53, p16, Sirt1 in the USP22−/− and the parent cancer cells

One of crucial functions of USP22 in cancers is to transcriptionally regulate gene expression through modulating H2Bub1 [7]. To define the biology and molecular mechanisms of USP22 in NSCLC, we performed RNA-seq to profile global gene expression change upon USP22 knockout in A549 (p53 wild-type, KRAS/G12S) and H1299 cancer cells (p53-null, NRAS/Q61K). RNAseq data analysis showed that more than 2000 genes are differentially expressed in two USP22−/− cancer cells (Additional file 1: Figure S1), and about 300 and 484 genes were unanimously upregulated or downregulated in two USP22−/− cancer cells (*P* < 0.01, false discovery rate: FDR < 0.05, log2 fold change: log2 FC ≥ 1, Additional file 2: Table S1). The original RNA-seq data is saved on NCBI GEO website with accession number GSE131934. Remarkably, USP22 knockout had pronounced effects on expression of these important cancer-associated gene sets such as c-Myc, E2F, and selected genes including ALDH1A3, CCNE1/G1, E2F6, HOXA1, MMP9, NFKB2, TP63, TPM4, SET7/9 were validated by qRT–PCR (Fig. 2a). Using gene ontology (GO) enrichment analysis, we identified that angiogenesis, cell cycle progression, epithelial-mesenchymal transition (EMT), KRAS, and c-Myc signaling pathways were significantly downregulated in the USP22−/− cancer cells (Fig. 2b). In contrast, processes related to phosphorylation and tight junction were significantly upregulated in the two USP22−/− cancer cells (Fig. 2b). In line with RNA-seq and Go analysis, Western blot analysis validated these changes in gene expression, and revealed a moderate increase of E-cadherin and/or decrease of Vimentin in USP22−/− cancer cells that reflects suppression of EMT singaling pathway (Fig. 2c). Furthermore, we also found that USP22 knockout drastically suppressed activation of AKT, ERK signaling pathways in both A549 and H1299 cancer cells (Fig. 2c). In addition, a previous study demonstrated that USP22 may suppress p53 function through deubiquitinating and stabilizing Sirt1 [22]. However, we found that both Sirt1 and p53 protein were not dramatically changed in the p53-wildtype A549 cancer cells (Fig. 2d), indicating p53 may not play a decisive role in USP22-mediated malignancy in NSCLC. We also found that USP22 knockout affected expression of genes that regulate cell cycle progression, and decreased cyclin D1/2 and E1 proteins (Fig. 2d); and moderately elevated cyclin-dependent kinase (CDK) inhibitor p16, which also play a critical role in cell cycle progression at G1 and S phase (Fig. 2d). It should be pointed out that not all of differentially expressed proteins upon USP22 knockout are regulated through transcriptional mechanisms. In summary, these data indicate that USP22 knockout affects multiple pathways involved in NSCLC progression.

### USP22 knockout significantly suppresses in vivo growth of NSCLC cells

**Fig. 3:**
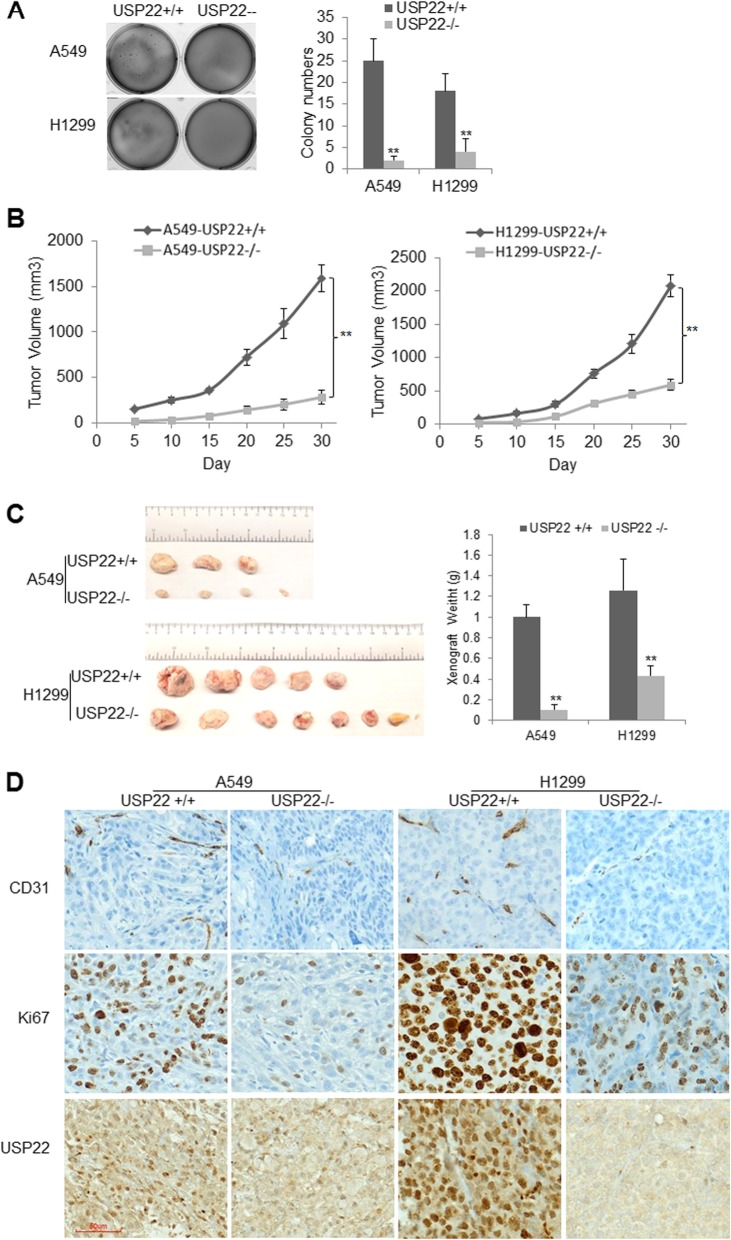
USP22 knockout suppresses angiogenesis and growth of A549 and H1299 cells. **a**. Colony formation assays, results show colonies formed within 3 weeks, compared to their parent cells, ** *P* < 0.01. **b.** The in vivo growth volumes of the USP22−/− and the parent A549 and H1299 cells (USP22−/− versus USP22+/+, ** *P* < 0.01). **c**. Represent images and average weights of xenografts (USP22−/− versus USP22+/+, ** *P* < 0.01). **d**. IHC stains for endothelial cell marker CD31, proliferation marker Ki67, and USP22 in xenografts generated by the USP22−/− and the parent USP22+/+ cancer cells (magnification, × 200)

To investigate its effects on in vitro proliferation and survival in NSCLC, we first knocked down USP22 by siRNA in A549 and H1299 cells. Consistent with our previous study [11], we found that transient USP22 knockdown slightly increased H2Bub1 and trimethylation of both H3K79 and H3K4 levels in these cells (Additional file 1: Figure S2A), and USP22 knockdown significantly inhibited in vitro proliferation of both A549 and H1299 cells (Additional file 1: Figure S2B), and induced a moderate G1-S phase arrest independent of their p53 status, but barely induced apoptosis (Additional file 1: Figure S2C-D). We then compared the colony formation and in vivo growth of USP22−/− cancer cells with their parent cells. We found that USP22−/− A549 and H1299 cancer cells generated much fewer and smaller colonies within 3 weeks compared to their parent cancer cells (Fig. 3a). Xenograft experiments further revealed that the growth and volume of USP22−/− A549 and H129 were significantly less than their parent cells (*N* = 8) in NSG mouse (Fig. 3b, *P* < 0.01). And at the end of experiment, xenograft weights of USP22−/− cancer cells were much more significantly less than their parent cancer cells (Fig. 3c, *P* < 0.01). The pronounced suppression of xenograft growth by USP22 knockout was further supported by immunostaining of Ki67 (a proliferation marker), which showed that the intensity of Ki67 immunostaining and percentage of Ki67-positive cells were much lower in USP22−/− cancer cell xenografts than their parent cancer cells (Fig. 3d, upper panel). To investigate the effect of USP22 knockout on angiogenesis, the blood vessel density was analyzed by quantifying immunostaining of CD31 (an endothelial cell marker). The results showed that CD31 immunostainings were much lower in xenografts generated by USP22−/− cancer cells than their parent cancer cells (Fig. 3d, upper panel), indicating that in vivo angiogenesis was dramatically suppressed upon USP22 knockout. Additionally, the USP22 nuclear immunostaining was only found in the parent cancer cell xenografts but not in USP22−/− cancer cell xenografts (Fig. 3d, upper panel) and adjacent normal cells and tissues (Additional file 1: Figure S3). Therefore, these data demonstrated that the USP22 knockout significantly suppresses in vivo cancer growth of NSCLC.

### USP22 knockout inhibited in vivo metastasis of NSCLC and prolonged the survival of metastatic cancer-bearing NSG mice

**Fig. 4:**
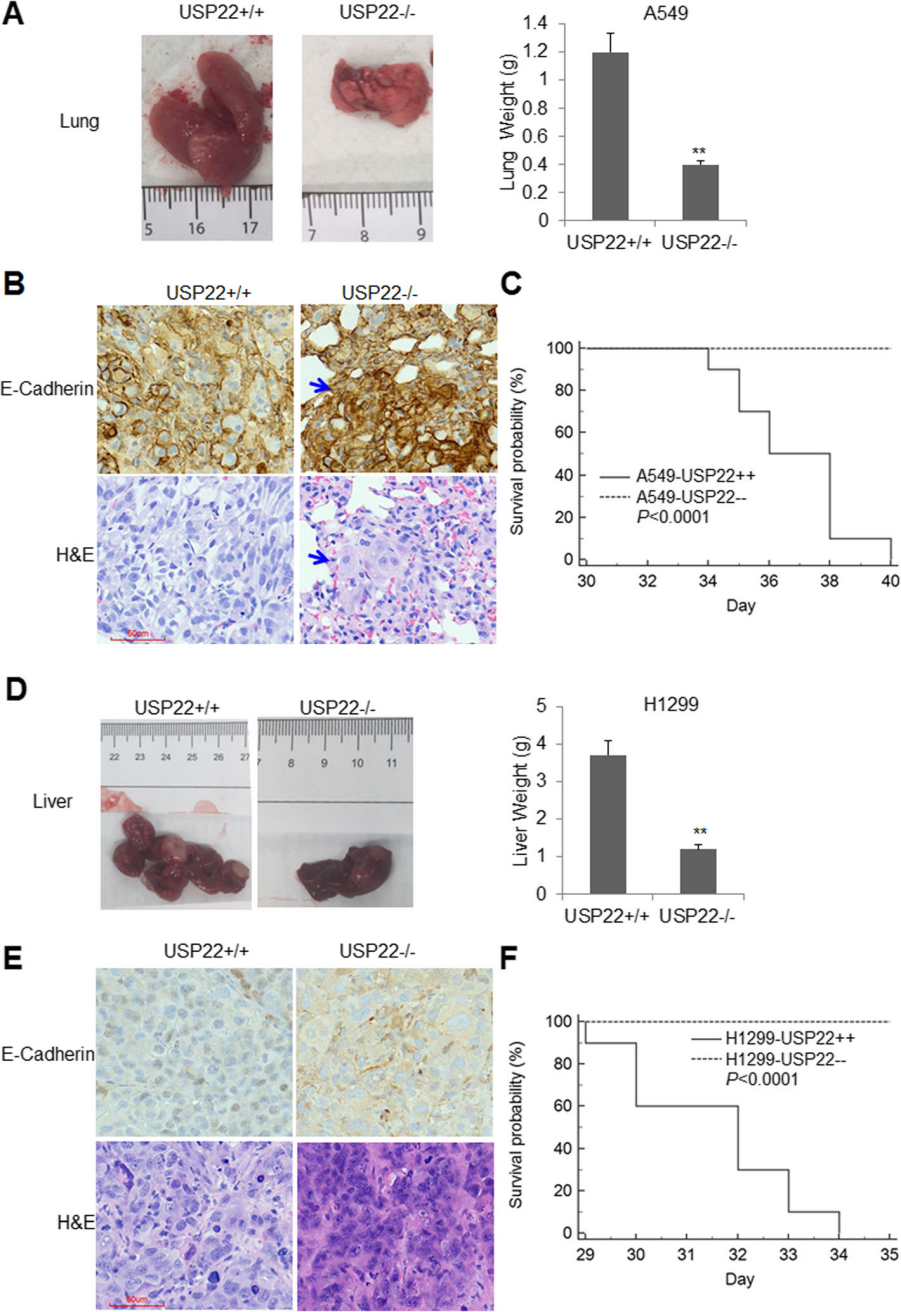
Effects of USP22 knockout on metastasis and survival of metastatic cancer-bearing mice. **a**. NSG mice were injected with 2 × 10^6^ of the parent (USP22+/+) or USP22−/− A549 cancer cells (*n* = 10) through tail vein. Four weeks after injection, mice were killed, lungs (left panel) were excised and weighted (right panel), ** *P* < 0.01 compared with A549 cells. **b**. E-cadherin IHC and H&E stains of lung metastasis formed by A549 (USP22+/+) (left panel) or USP22−/− cancer cells. H&E shows almost 100% area of lung was occupied by A549 metastasis, while less than 5% (blue arrow area) was occupied by USP22−/− A549 cancer cells (magnification, × 200). **c.** Kaplan-Meier survival curves of mice bearing the parent and USP22−/− cancer cells (*P* < 0.0001)*.* **d**. NSG mice were injected with 2 × 10^6^ of the parent (USP22+/+) or USP22−/− H1299 cancer cells (n = 10) through tail vein. Four weeks after injection, mice were killed, livers (left panel) were excised and weighted (right panel), ** *P* < 0.01 compared with the parent H1299 cancer cells. **e.** E-cadherin IHC and H&E stains of lung metastasis formed by the parent (left panel) or USP22−/− H1299 cancer cells (right panel) (magnification, × 200). **f.** Kaplan-Meier survival curves of mice bearing the parent and USP22−/− H1299 cancer cells, ** *P* < 0.01 compared with the parent H1299 cancer cells

We first measured the in vitro invasive potentials of A549 and H1299 cells upon USP22 knockout, and found that the migration (Additional file 1: Figure S4A) and invasion (Additional file 1: Figure S4B) of USP22−/− cancer cells were significantly decreased compared with their parent cells. We then assessed the in vivo metastasis of NSCLC cells using mouse lung and liver metastasis models by injecting 2 × 10^6^ cancer cells into the tail veins of 8–10 week-old NSG mice. About 4 weeks later, the cancer-bearing mice were euthanized, and both liver and lung metastasis were evaluated by counting visible tumor nodules per mouse and the total weight of excised lung and liver. Interestingly, A549 cancer cell, which was derived from a primary lung adenocarcinoma, formed visible metastasis in lung only. Metastatic cancer nodules (about 8–11/each) were found in 100% (8/8) of mice injected with A549 cells, and metastatic cancers diffusely filled in the entire lung (Fig. 4a, left panel). In contrast, none of mice (0/10) injected with USP22−/− A549 cells developed visible cancer nodules (Fig. 4a, left panel). And the average weigh of lungs excised from the mice injected with USP22−/− cancer cells was significantly lighter than that of A549 cells (Fig. 4a, right panel). And hematoxylin and eosin stain (H&E) stain of the excised lung tissues further shows A549 cancer cells almost filled the whole lung, while USP22−/− A549 cancer cells only formed micro-metastasis (Fig. 4b, lower panel). Since EMT and decreased E-Cadherin may enhance invasion and metastasis [23], therefore, we further measured E-Cadherin in this metastasis. Consistently with above metastasis data, IHC analysis further uncovered that E-Cadherin protein were dramatically upregulated in USP22−/− cancer xenografts compared to the parent A549 cancer xenografts (Fig. 4b, upper panel). In a separate experiment, we investigated the survival of NSG mice injected with USP22−/− and the parent A549 cancer cells. The animals were observed daily until their death. All of 10 (100%) mice injected with the parent A549 cancer cells died of metastatic cancers by day 40; in contrast, all of the mice injected with USP22−/− A549 cancer cells were alive at day 35, and Kaplan-Meier survival analysis showed the survivals of USP22−/− A549 cancer-bearing mice were significantly longer than that of A549 cancer-bearing mice (*P* < 0.0001, Fig. 4c).

Interestingly, H1299 cancer cell, which was derived from a lymph node metastasis of lung adenocarcinoma, dominantly developed metastatic cancer in liver of NSG mouse. As shown in Fig. 4d, compared with the metastatic cancer nodules (having a clear boundary) formed by H1299 cancer cells (5–8 large nodules/each), the metastatic cancer nodules formed by USP22−/− H1299 cancer cells were much less abundant and smaller (1–3 small nodules/each). IHC analysis shows that E-cadherin protein also dramatically upregulated in USP22−/− H1299 xenograft tissues (Fig. 4e). The Kaplan-Meier analysis shows that USP22 knockout significantly prolonged survival of metastatic cancer-bearing mice (Fig. 4f, *P* < 0.0001). Therefore, all data demonstrated that USP22 knockout significantly suppressed metastasis of NSCLC, and prolonged survival of metastatic cancer-bearing mice.

### USP22 knockout impairs non-homologous DNA damage repair and enhances cisplatin sensitivity in NSCLC cells

**Fig. 5:**
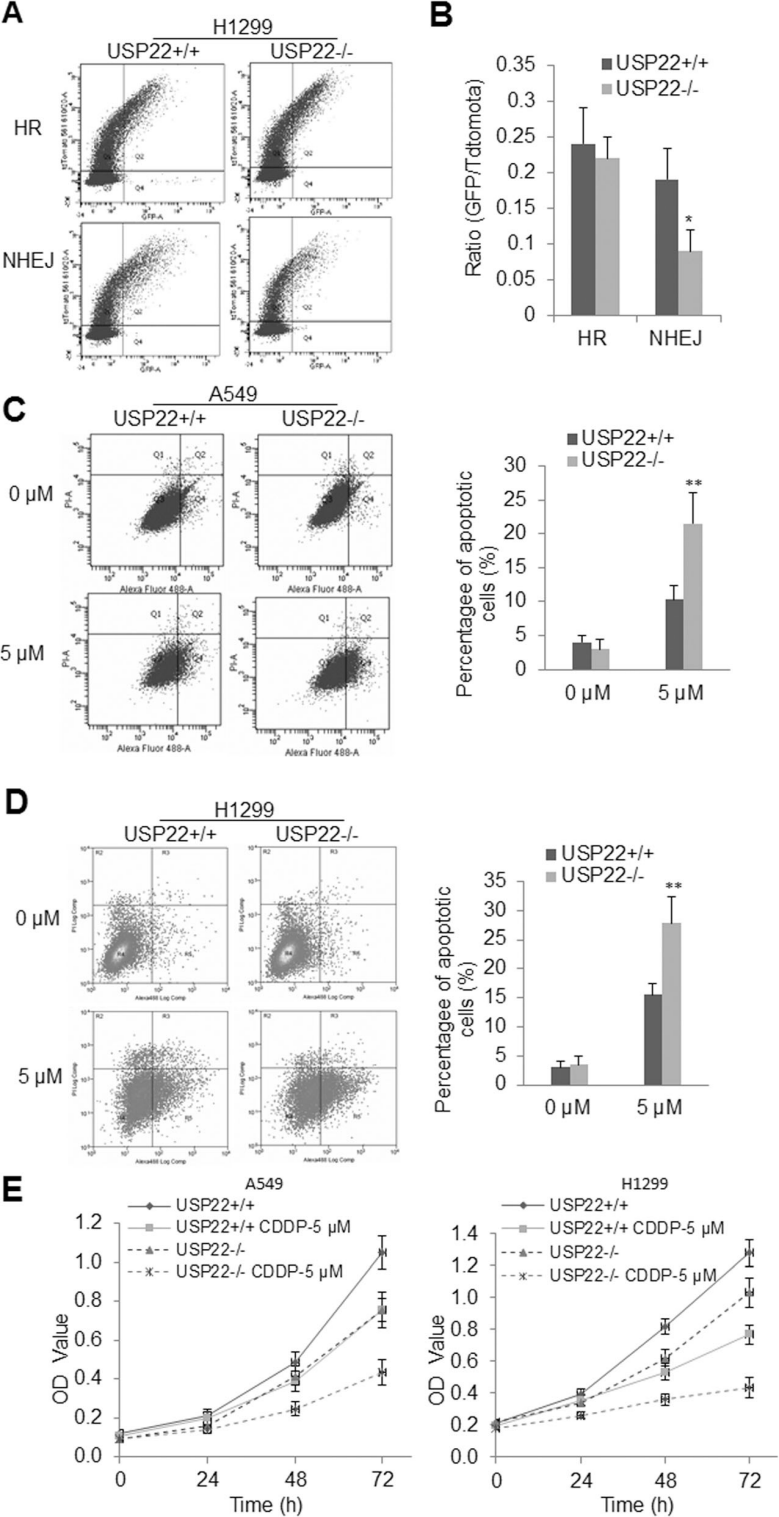
USP22 knockout impairs NHEJ in NSCLC cells and sensitizes cancer cells to cisplatin treatment. **a**. Representative flow cytometry charts of the parent and USP22−/−H1299 cancer cells for HR and NHEJ reporter assays. **b**. Quantitative analysis of HR and NHEJ reporter; * *P* < 0.05, USP22−/− versus USP22+/+. Error bars are representative of three individual treated samples of two experimental duplicates. **c**. Representative flow cytometry apoptotic profiles of the parent A549 versus USP22−/− A549 (left panel) and the quantitative analysis of apoptotic cells (right panel) showing apoptotic cells were significantly increased in USP22−/− A549. **d**. Representative flow cytometry apoptotic profiles of the parent H1299 versus USP22−/− H1299 cancer cells (left panel) and the quantitative analysis of apoptotic cells (right panel) showing apoptotic cells were significantly increased in USP22−/− H1299 cells. The experiment was repeated three times and data represent the average of the early apoptotic and late apoptotic cells; ** *P* < 0.01. **e.** The in vitro proliferation curves of the parent A549 and A549-USP22−/− (left panel); the parent H1299 and H1299-USP22−/− cancer cells (right panel) over a period of 72 h of cisplatin (CDDP) treatment, cells were treated with 5 μM cisplatin and proliferation of each cell were measured by Kit-8 at 24 h, 48 h and 72 h post-treatment and presented as the averages of OD values of triplicated experiments

A previous study demonstrated that the SAGA deubiquitination module promotes DNA repair [24, 25]. In addition, we recently found that expression of USP22 is associated with cisplatin resistance in cancer-initiating cells (CIC) from primary lung adenocarcinoma [12]. Consistently, we herein identified that USP22 is drastically upregulated in A549 and H1299 cancer cells that survived cisplatin treatment **(**Additional file 1: Figure S5), indicating an involvement of USP22 in cisplatin resistance and DNA damage repair. To further explore the therapeutuic application of targeting USP22 and underlying mechanisms, we examined the impact of USP22 knockout on HR or NHEJ repairs for DNA double-strand breaks (DSB) in NSCLC. Using the reporter assays of DR-GFP for HR and EJ5-GFP for NHEJ as described previously [21], we found that USP22 knockout did not significantly affect HR potential (Fig. 5a upper panel, Fig. 5B), but significantly inhibited the NHEJ efficacy in H1299 cells (Fig. 5a lower panel, Fig. 5b), which was presented as decreased ratio of GFP-positive cell to tdTomato fluorescent protein-positive cell that was used as the control for transfection efficacy. We herein measured apoptosis in both the parent and USP22−/−cells treated with 5 μM cisplatin for 72 h by Annexin-V flow cytometry analysis of apoptotic cells. Representative flow cytometry plots show that both USP22−/− A549 (Fig. 5c, left panel) and USP22−/− H1299 cells (Fig. 5d, left panel) were significantly more sensitive to cisplatin than their parent cells (Fig. 5c-d, right panel, *P* < 0.01), indicating USP22 knockout sensitized these two cells to cisplatin treatment. In addition, the in vitro proliferation assays further revealed that both A549-USP22−/− (Fig. 5e, left panel) and H1299-USP22−/− cancer cells (Fig. 5e, right panel) were more sensitive to 5 μM cisplatin than their parent cells over a period of 72 h of treatment. And at 72 h post-treatment, the percentages of viable USP22−/− A549 and USP22−/− H1299 cells treated with 5 μM cisplatin was around 45 and 35%, while the percentages of their parent cells treated with 5 μM cisplatin were around 70 and 55% of their untreated control cells, respectively.

### USP22 knockout sensitize RAS-mutant lung cancer cells to irradiation

**Fig. 6:**
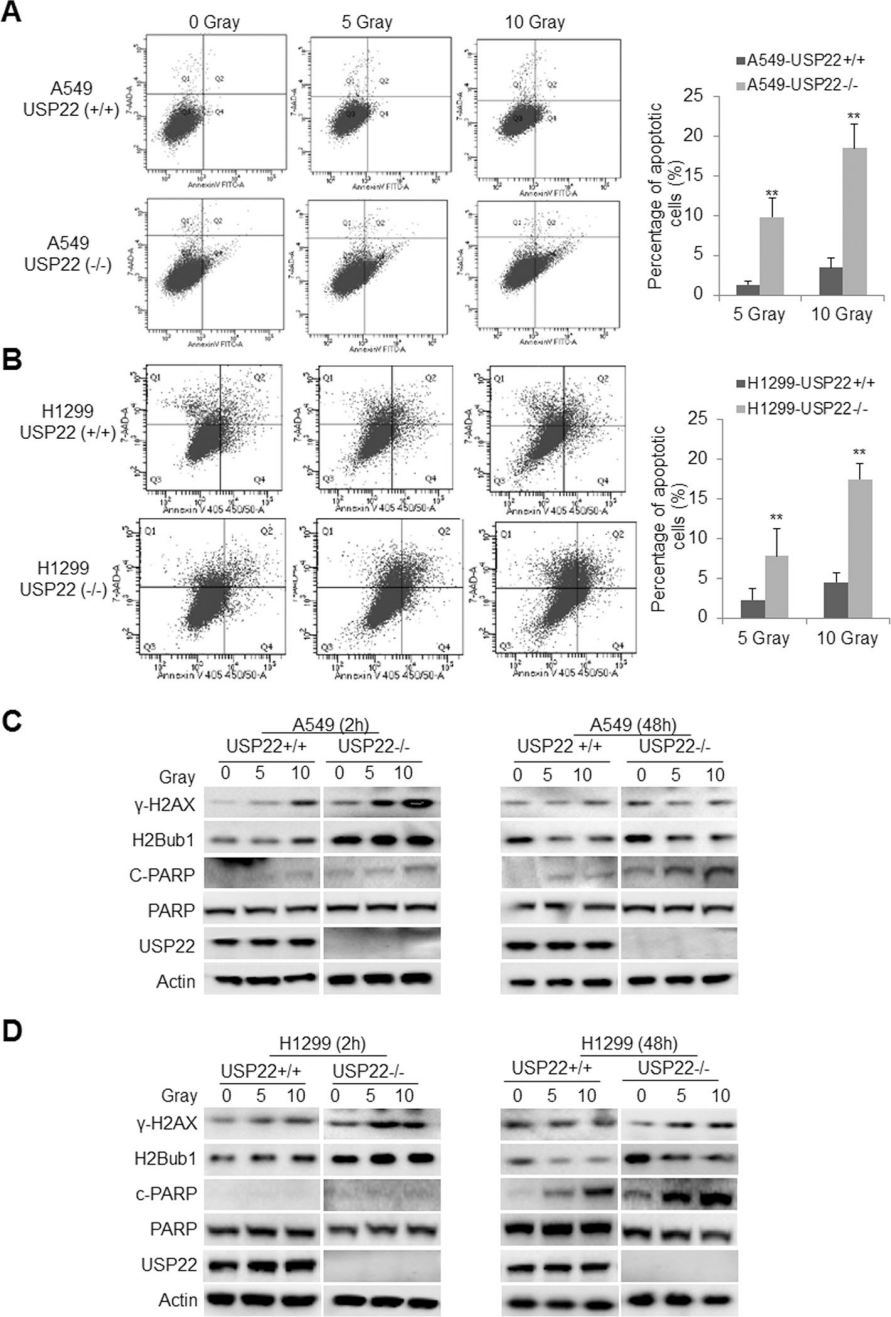
USP22 knockout sensitizes NSCLC cells to irradiation. Representative flow cytometry profile of apoptotic cells in **a**. The parent (upper panel) and USP22−/− A549 (lower panel) cancer cells; and **b**. The parent (upper panel) and USP22−/− H1299 cancer cells (lower panel). Cells were first subjected to 5 or 10 Gy irradiation; apoptosis was measured at 48 h post-irradiation. Quantitative analysis of the experiments shows that apoptotic cells were significantly increased in both USP22−/− A549 (**a**, right panel) and USP22−/− H1299 cancer cells (**b**, right panel) compared with their parent cells (USP22+/+). The experiment was repeated three times and data represent the average of the early apoptotic and late apoptotic cells (** *P* < 0.01). The dynamics of γ-H2AX, H2Bub1, and apoptotic markers PARP cleaved product (c-PARP for cleaved protein) in **c**. the parent and USP22−/− A549 cancer cells, and **d**. the parent and USP22−/− H1299 cancer cells at 2 h, 48 h post-irradiation that analyzed by Western blot

Since USP22 knockout significantly decreased the capacity of NHEJ, a major pathway for DNA DSBs repair, we further investigated whether USP22−/− cancer cells are more sensitive to irradiation that causes DSBs for cancer treatment. Apoptosis analysis showed that both 5 and 10 Gy irradiation induced more apoptosis at 48 h post-irradiation in both USP22−/− A549 (Fig. 6a, lower panel) and USP22−/− H1299 (Fig. 6b, lower panel) cancer cells than the parent A549 (Fig. 6a, upper panel) and H1299 (Fig. 6b, upper panel) cancer cells, respectively. Statistical analysis further revealed a significant increase in apoptotic cells in USP22−/− than their parent cells (Fig. 6a-b, right panel). Moreover, by Western blot, we also tracked the dynamics of γ-H2AX, a marker for DNA damage, to measure the extent of DNA damage and the speed of damage repair; and found that irradiation elevated γ-H2AX and H2Bub1 proteins in both the parent and USP22−/− cancer cells, while more γ-H2AX and H2Bub1 were induced in USP22−/− A549 cells (Fig. 6c, left panel) and USP22−/− H1299 cells (Fig. 6d, left panel) than their parent cells at 2 h post-irradition. And a slightly more γ-H2AX and H2Bub1 were still present in these USP22−/− H1299 cancer cells at 48 h post-irradition (Fig. 6c-d, right panel), indicating USP22 knockout impaired deubiquitination of H2Bub1 that may be required for prompt and correct DSB repair. Consistently, much more cleaved-PARP were found in USP22−/− cells (especially in USP22−/− H1299) than their parent cancer cells (Fig. 6c-d, right panel) at 48 h post-irradition, indicating more apoptotic cancer cells were induced. We also compared the dynamics of p53 protein in USP22−/− and the parent A549 cells after irradiation treatment, the results show only a slightly more p53 protein was found in USP22−/− A549 cells at 6, 12 h post-irradiation (Additional file 1: Figure S6), indicating p53 may not play a crucial role in the process for USP22−/− cancer cells. Taken together, the above data strongly suggest that USP22 knockout can significantly enhance irradiation induced-apoptosis in NSCLC cells.

### USP22 is associated with cancer angiogenesis in NSCLC

**Fig. 7:**
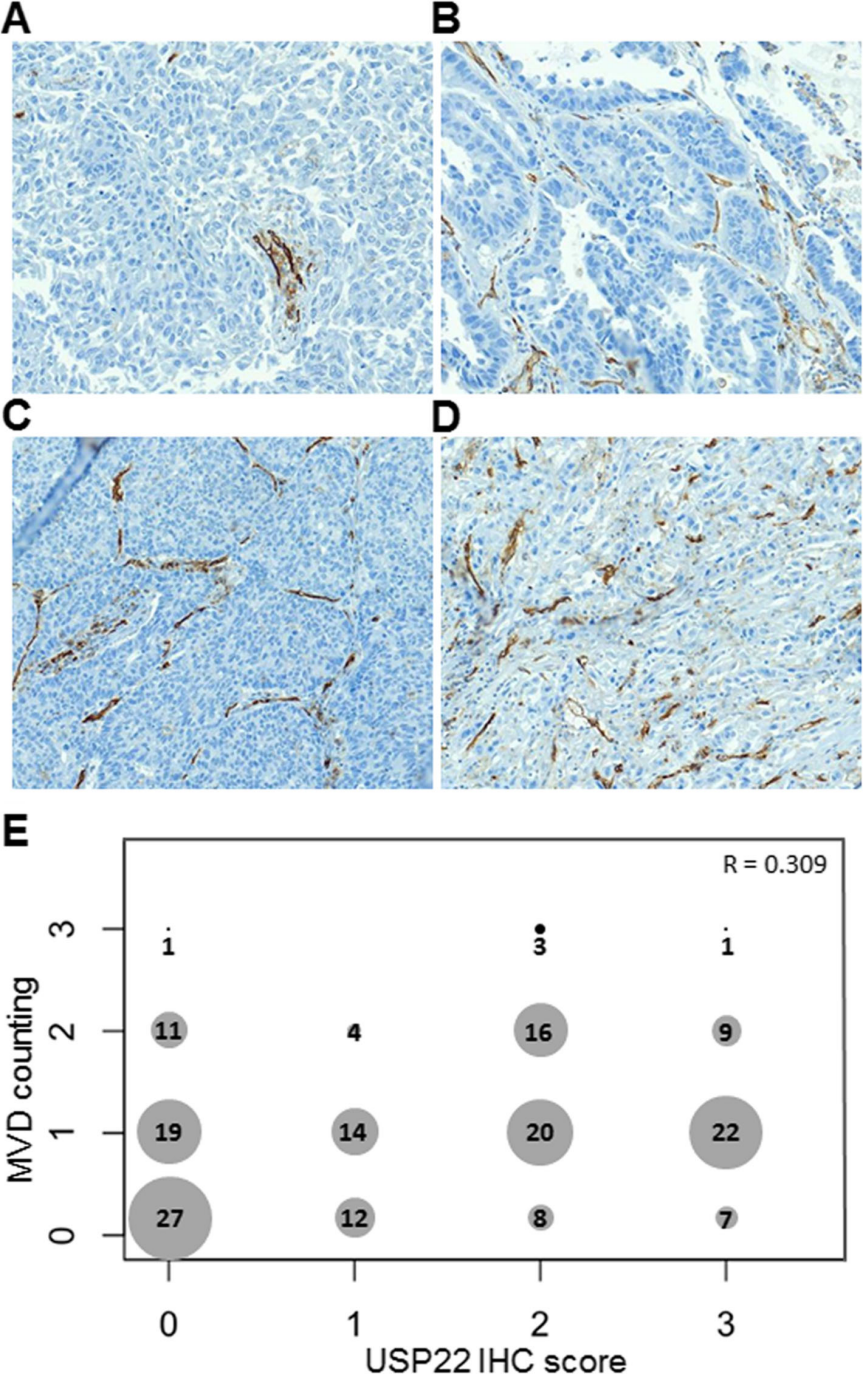
USP22 is associated with enhanced cancer angiogenesis in NSCLC. **a**. Photomicrographs of four representative NSCLC sections with rare (R), low (L), medium (M), and high (H) MVD measured by CD31 staining (magnification, × 100). **b**. Cross distribution and correlation between USP22 immunostaining score and MVD counting in 174 NSCLC tissues. X axis is for USP22 IHC score, Y axis is for MVD counting. Number in dot is the sample size with various USP22 and MVD. R is the Spearman’s correlation coefficient for USP22 and MVD

Both RNAseq analysis and in vivo xenograft experiments demonstrated that USP22 may play an important role in cancer angiogenesis. We next investigated the correlation between USP22 and MVD in NSCLC tissues. Figure 7 shows the representative tissue sections with rare (R/0, Fig. 7a), low (L/1+, Fig. 7b), medium (M/2+, Fig. 7c), and high (H/3+, Fig. 7d) MVD were revealed by endothelial cellular marker CD31 IHC staining. Using Spearman’s nonparametric correlation analysis, we identified a moderately positive correlation between the scores of USP22 immunostaining and MVD in 174 NSCLC tissues (Fig. 7d, *R* = 0.309, *P* < 0.001). This data further indicates that higher USP22 may promote angiogenesis in NSCLC.

## Discussion

Overexpression of USP22 protein has been reported in numerous cancers including lung adenocarcinoma, and is associated with poor prognosis of cancer patients [6, 8–10, 26]. For example, in stomach cancer, increased USP22 in cancer tissues was associated with shorter patient survival [27]. Unfortunately, studies also suggest USP22 may function as a tumor suppressor in cancer due to its function in genome stability and frequent loss (both homozygous or heterozygous loss) and downregulation in many cancer types including ovarian, esophagus, colorectal, pancreatic, lung adenocarcinoma, breast and stomach cancers [14, 15]. Accordingly, these data suggest that USP22 may be a haplo-insufficient tumor suppressor gene, whose diminished expression may also contribute to cancer development. Surprisingly, a very recent study demonstrated that USP22 deficiency leads to myeloid leukemia upon oncogenic KRAS activation through a PU.1 dependent mechanism [16]. Therefore, the roles of USP22 in initiation and development of various human cancers remain to be elucidated. In lung cancer, USP22 was reported to be correlated with advanced differentiation and stage, poor prognosis of NSCLC cancer [26]. In this study, we also found that USP22 is upregulated in lung adenocarcinoma; and upregulation of USP22 protein was more frequently found in advanced stage and was associated with the recurrence of NSCLC, indicating USP22 plays oncogenic roles in NSCLC.

Several earlier studies indicated that within SAGA complex USP22 deubiquitylates H2Bub1, where it is required for transcription [7]; and ablation of Usp22 in primary B cells results in elevated both baseline and irradiation induced H2Bub1 [24, 25]. However, some other studies also indicate that USP22 loss does not significantly increase globale H2Bub1 levels. For example, a study showed that knockout of Usp22 significantly influenced the frequency of differentiated cells in the small intestine and the brain, while H2Bub1 levels remained constant [28]. Another study unexpectedly demonstrated that ablation of USP22 leads to a reduction, rather than an increase, in global H2bub1 levels in HEK 293 T and H116 colorectal cancer cells [29]. Although we observed that knockout of USP22 didn’t lead to a significant increase of global H2Bub1 in two NSCLC cell lines, H2Bub1 protein might locally increase at loci of a subset of genes that are transcriptionally regulated by USP22. Moreover, it is also reported that H2Bub1 is quite dynamic, as it disappears only minutes after transcription is blocked [30]; and the status of cells in cell-cycle phase, activity of ubiquitinase RNF20/RNF40 complex, and other deubiquitinases may also significantly impact on H2Bub1 protein levels in different cells upon USP22 knockout. In addition, it should be noted that USP22 regulates other targets through post-transcriptional mechanisms. Although more investigation is required to further explore if and how USP22 exactly modulates H2Bub1 protein in various mammalian cells, it is clear that both USP22 and H2Bub1 are centrally involved in gene transcriptional regulation [7, 31–35]. The precise mechanisms through which USP22 affects cancer progression are largely unknown in lung adenocarcinoma. By using RNAseq and Go ocology enrichment analysis, we found that USP22 knockout had pronounced effects on the expression of these important cancer-associated genes including ALDH1A3, CCND1/2, CCNG1, Set7/9, c-Myc etc., significantly suppressed angiogenesis, cell cycle progression, EMT, RAS, c-Myc signaling pathways; and concurrently enhanced oxidative phosphorylation and tight junction signaling pathways from the Kyoto Encyclopedia of Genes and Genomes (KEGG) in the two cells. And an earlier study reported that USP22 is required for activated transcription and cell-cycle progression, and critical for cell proliferation [7]. Another previous study showed that c-Myc was regulated by H2Bub1 [35]. In agreement with these findings, we found that USP22 knockout significantly upregulated gene expression of these growth-promoting oncogenes including, c-Myc, cyclins, and E2F1/2. And compared with the parent cells, USP22 knockout induced cell-cycle arrest and significantly suppressed both in vitro and in vivo of lung adenocarcinoma growth [13]. Oncogenic roles of USP22 in cancers partially ascribes to its regulation on c-Myc oncogene transcriptional activity [7]. In this study, by RNAseq and gene set enrichment analysis, we also found that USP22 knockout moderately decreased c-Myc protein and its signaling transduction. It is worthwhile to point out that USP22 may also impact other molecules involved in proliferation and cell cycle progression such as cyclin B2 [36] and cyclin D1 [37]. We herein also find that cyclin E and E2F1 signal pathway may also contribute to proliferation-promoting effect of USP22. In addition, a previous study indicated that USP22 may suppress p53 function through deubiquitinating and stabilizing Sirt1 [22]. However, we found that both Sirt1 and p53 protein were not significantly changed in the p53-wild-type A549 cancer cells, indicating this pathway may not play a crucial role in context of lung cancer. By the RNAseq analysis, we found that USP22 knockout significantly suppressed angiogenesis and EMT signaling pathways that play essential roles in cancer growth and metastasis. Angiogenesis is a critical and rate-limiting step in tumor progression [38], and angiogenesis is also essential for the dissemination and establishment of tumor metastases [39, 40]. Many clinical studies on NSCLC have revealed that angiogenesis and MVD is closely correlated with tumor growth and postoperative prognosis of cancer patients [41, 42]. Interestingly, by compared the in vivo angiogenesis in xenografts of USP22 knockout and the parent cancer cells, we confirmed that USP22 knockout significantly suppresses angiogenesis. And RNAseq revealed that multiple genes involved in angiogenesis such as HIF1a, VEGF etc. were significantly downregulated in USP22 knockout cancer cells. Notably, through analysis the correlation of USP22 and MVD in NSCLC tissue samples, we have found a significantly positive correlation between these two stainings, indicating that elevated USP22 also promotes angiogenesis in cancer. And this finding is consistent with that of a very recent study that demonstrated that USP22 controls multiple signaling pathways that are essential for vasculature formation in the mouse placenta [43]. Furthermore, EMT is known to be a central mechanism responsible for invasiveness and metastasis of various cancers [44]. We herein first found that tight junction gene set were upregulated in USP22 knockout cancer cells, and further identified that compared with the parent cells, the cell-cell adhesion receptor E-cadherin was upregulated in the two USP22 knockout cells, and this upregulation was more pronounced in in vivo metastatic tumors. Consistently, in vivo metastatic model of lung cancer revealed that USP22 knockout cancer cells formed a drastically less and smaller metastatic cancers in liver and lung, and significantly prolonged the metastatic cancer-bearing mice through modulation of these invasion and metastasis related genes such as E-cadherin. It has long been recognized that E-cadherin is an important determinant of tumor progression, serving as a suppressor of invasion and metastasis in many contexts. Therefore, the above data indicates that targeting USP22 may significantly suppress in vivo metastasis of lung adenocarcinoma. H2Bub1 plays an important role in DNA damage checkpoint activation and timely initiation of repair [45, 46]. A previous study revealed that RNF20/RNF40 dependent H2Bub1 is needed for recruitment of repair factors in an ATM dependent manner and is necessary for faithful repair through both HR and NHEJ pathways [45]. Knockdown of RNF20/40 was shown to not affect formation of γH2AX foci but rather their persistence [45]. Interestingly, deubiquitination of H2Bub1 was also shown to act downstream of ATM and to facilitate formation of γH2AX foci through both HR and NHEJ [24, 25]. Using HR and NHEJ reporter system, we herein found that USP22 knockout significantly impaired NHEJ repair potential in lung adenocarcinoma cells. The underlying mechanism may be associated with H2Bub1 dynamics and γH2AX foci formation. On the other hand, the defect in DNA damage repair in USP22 knockout cancer cell may be exploited to gain synthetic lethality therapy in combination of DNA damage causes such as irradiation, similar to the BRCA-PARP synthetic lethality, which show that PARP inhibitors effectively kill tumors defective in the breast cancer gene 1 and 2 (BRCA1/2) through the concept of synthetic lethality [47]. Notably, we herein found that compared with the parent cancer cells, USP22 knockout cell were much more sensitive to irradiation, indicating therapeutic implication of targeting USP22 as an approach to combine with other conventional treatment. One of the hallmarks of cancer is acquisition of resistance to apoptosis, resulting in cells refractory to therapy [38]. Lung cancer cells are associated with resistance to drug-induced apoptosis, in particular to platinum chemotherapy, the most commonly used chemotherapeutic for lung cancer [48, 49]. USP22 was also previously reported to be associated with chemo resistance, and knockdown of USP22 sensitized through suppression on PI3K-AKT signaling pathway [50]. We recently showed that USP22 may be associated with cisplatin resistance in lung cancer stem cell through downregulation of ALDH1A3 [12]. We here also found that p-AKt signaling pathway is also suppressed in USP22 knockout cells and USP22 knockout cells are more sensitive to cisplatin treatment. Indicating USP22 may cause drug resistance through multiple targets or signaling pathways. Therefore targeting USP22 may sensitize cancer cells to irradiation and cisplatin treatment.

## Conclusions

In summary, overexpression of USP22 is found in a half of 202 NSCLC tissues; and high USP22 is associated with NSCLC recurrence. USP22 knockout dramatically suppressed in vivo angiogenesis*,* growth, and metastasis of NSCLC xenografts independent of their p53 status, and significantly prolonged survival of metastatic cancer-bearing mice. Furthermore, USP22 knockout impaired non-homologous DNA damage repair capability, significantly enhanced cisplatin- and irradiation-induced apoptosis in the cells. Therefore, our findings strongly suggest that USP22 plays critical oncogenic roles in the malignancy and progression of NSCLC and provide rationales for targeting USP22, which may induce broad anti-cancer activities via suppressing multiple signaling pathways including angiogenesis, EMT, c-Myc, and KRAS, as a novel therapeutic strategy for NSCLC.

## Supplementary information

**Additional file 1 Fig. S1.** Differentially expressed gene in USP22-Ko A549 and H1299 cells. **Fig. S2.** Impact of knockdown of USP22 on H2Bub1/methylatiom of H3K4/K79, cancer cell proliferation, and cell cycle progression. **Fig. S3**. Negative USP22 IHC stains in adjacent normal cells and tissues. **Figure** 4. Impact of knockout of USP22 on in vitro migration and invasion of A549 and H1299 cells. **Fig. S5.** Elevated USP22 in cancer cells survived cisplatin treatment. **Fig. S6.** Dynamics of P53 in A549 and USP22-Ko cells after 5 and 10 Gy irradiation.

**Additional file 2.** Differentially expressed gene list in USP22-Ko A549 and H1299 cells.

## Data Availability

The dataset supporting the conclusions of this article is included within the article and its additional file. RNA-Seq data is saved on NCBI GEO website with accession number GSE131934.

## Abbreviations

ALDH1A3: Aldehyde dehydrogenase family 1 member A3
BRCA: breast cancer gene
CCNE/G: cyclin E/G
CDK: cyclin-dependent kinase
EGFR: Epidermal growth factor receptor
EMT: Epithelial-to-mesenchymal transition
ERK: Extracellular signal-regulated kinase
FGFR: fibroblast growth factor receptor
H2Bub1: H2B monoubiquitination
HOXA9: Homeobox protein Hox-A9
HR: homologous repair
IHC: Immunohistochemistry
MMP9: Matrix Metallopeptidase 9
NHEJ: non-homologous end joining
NSCLC: Non-small cell lung cancer
PARP: Poly (ADP-ribose) polymerase
SAGA: Spt-Ada-Gcn5-Acetyltransferase
SIRT1: NAD-dependent deacetylase sirtuin-1
TPM4: tropomyosin 4
USP22: ubiquitin-specific protease 22
γH2AX: Phosphorylation of the Ser-139 residue of the histone H2AX

## References

[1] Ferlay J, Shin HR, Bray F, Forman D, Mathers C, Parkin DM (2010). Estimates of worldwide burden of cancer in 2008: GLOBOCAN 2008. Int J cancer J Int Du cancer.

[2] Chen Z, Fillmore CM, Hammerman PS, Kim CF, Wong KK (2014). Non-small-cell lung cancers: a heterogeneous set of diseases. Nat Rev Cancer.

[3] Morgensztern D, Campo MJ, Dahlberg SE, Doebele RC, Garon E, Gerber DE, Goldberg SB, Hammerman PS, Heist RS, Hensing T (2015). Molecularly targeted therapies in non-small-cell lung cancer annual update 2014. J Thor Oncol.

[4] Siegel R, Ma J, Zou Z, Jemal A (2014). Cancer statistics, 2014. CA Cancer J Clin.

[5] Wilting RH, Dannenberg JH (2012). Epigenetic mechanisms in tumorigenesis, tumor cell heterogeneity and drug resistance. Drug Resist Updat.

[6] Cole AJ, Clifton-Bligh R, Marsh DJ (2015). Histone H2B monoubiquitination: roles to play in human malignancy. Endocr Relat Cancer.

[7] Zhang XY, Varthi M, Sykes SM, Phillips C, Warzecha C, Zhu W, Wyce A, Thorne AW, Berger SL, McMahon SB (2008). The putative cancer stem cell marker USP22 is a subunit of the human SAGA complex required for activated transcription and cell-cycle progression. Mol Cell.

[8] Glinsky GV, Berezovska O, Glinskii AB (2005). Microarray analysis identifies a death-from-cancer signature predicting therapy failure in patients with multiple types of cancer. J Clin Invest.

[9] Zhang Y, Yao L, Zhang X, Ji H, Wang L, Sun S, Pang D (2011). Elevated expression of USP22 in correlation with poor prognosis in patients with invasive breast cancer. J Cancer Res Clin Oncol.

[10] Liu YL, Yang YM, Xu H, Dong XS (2011). Aberrant expression of USP22 is associated with liver metastasis and poor prognosis of colorectal cancer. J Surg Oncol.

[11] Zhang K, Wang J, Tong TR, Wu X, Nelson R, Yuan YC, Reno T, Liu Z, Yun X, Kim JY (2017). Loss of H2B monoubiquitination is associated with poor-differentiation and enhanced malignancy of lung adenocarcinoma. Int J Cancer.

[12] Yun X, Zhang K, Wang J, Pangeni RP, Yang L, Bonner M, Wu J, Wang J, Nardi IK, Gao M (2018). Targeting USP22 suppresses Tumorigenicity and enhances Cisplatin sensitivity through ALDH1A3 Downregulation in Cancer-initiating cells from lung adenocarcinoma. Mol Cancer Res.

[13] Schrecengost RS, Dean JL, Goodwin JF, Schiewer MJ, Urban MW, Stanek TJ, Sussman RT, Hicks JL, Birbe RC, Draganova-Tacheva RA (2014). USP22 regulates oncogenic signaling pathways to drive lethal cancer progression. Cancer Res.

[14] Cerami E, Gao J, Dogrusoz U, Gross BE, Sumer SO, Aksoy BA, Jacobsen A, Byrne CJ, Heuer ML, Larsson E (2012). The cBio cancer genomics portal: an open platform for exploring multidimensional cancer genomics data. Cancer Discov.

[15] Jeusset Lucile, McManus Kirk (2017). Ubiquitin Specific Peptidase 22 Regulates Histone H2B Mono-Ubiquitination and Exhibits Both Oncogenic and Tumor Suppressor Roles in Cancer. Cancers.

[16] Melo-Cardenas J, Xu Y, Wei J, Tan C, Kong S, Gao B, Montauti E, Kirsammer G, Licht JD, Yu J (2018). USP22 deficiency leads to myeloid leukemia upon oncogenic Kras activation through a PU.1-dependent mechanism. Blood.

[17] Kosinsky RL, Zerche M, Saul D, Wang X, Wohn L, Wegwitz F, Begus-Nahrmann Y, Johnsen SA. USP22 exerts tumor-suppressive functions in colorectal cancer by decreasing mTOR activity. Cell Death Differ. 2019. 10.1038/s41418-019-0420-8 PMC7205880 31527800

[18] Zhang K, Wang J, Yang L, Yuan YC, Tong TR, Wu J, Yun X, Bonner M, Pangeni R, Liu Z (2018). Targeting histone methyltransferase G9a inhibits growth and Wnt signaling pathway by epigenetically regulating HP1alpha and APC2 gene expression in non-small cell lung cancer. Mol Cancer.

[19] Koukourakis MI, Giatromanolaki A, Thorpe PE, Brekken RA, Sivridis E, Kakolyris S, Georgoulias V, Gatter KC, Harris AL (2000). Vascular endothelial growth factor/KDR activated microvessel density versus CD31 standard microvessel density in non-small cell lung cancer. Cancer Res.

[20] Zhang K, Wang J, Wang J, Luh F, Liu X, Yang L, Liu YR, Su L, Yang YS, Chu P (2019). LKB1 deficiency promotes proliferation and invasion of glioblastoma through activation of mTOR and focal adhesion kinase signaling pathways. Am J Cancer Res.

[21] Zhang K, Keymeulen S, Nelson R, Tong TR, Yuan YC, Yun X, Liu Z, Lopez J, Raz DJ, Kim JY (2018). Overexpression of flap endonuclease 1 correlates with enhanced proliferation and poor prognosis of non-small-cell lung Cancer. Am J Pathol.

[22] Lin Z, Yang H, Kong Q, Li J, Lee SM, Gao B, Dong H, Wei J, Song J, Zhang DD (2012). USP22 antagonizes p53 transcriptional activation by deubiquitinating Sirt1 to suppress cell apoptosis and is required for mouse embryonic development. Mol Cell.

[23] Conacci-Sorrell M, Zhurinsky J, Ben-Ze'ev A (2002). The cadherin-catenin adhesion system in signaling and cancer. J Clin Invest.

[24] Ramachandran S, Haddad D, Li C, Le MX, Ling AK, So CC, Nepal RM, Gommerman JL, Yu K, Ketela T (2016). The SAGA Deubiquitination module promotes DNA repair and class switch recombination through ATM and DNAPK-mediated gammaH2AX formation. Cell Rep.

[25] Li C, Irrazabal T, So CC, Berru M, Du L, Lam E, Ling AK, Gommerman JL, Pan-Hammarstrom Q, Martin A (2018). The H2B deubiquitinase Usp22 promotes antibody class switch recombination by facilitating non-homologous end joining. Nat Commun.

[26] Ning J, Zhang J, Liu W, Lang Y, Xue Y, Xu S (2012). Overexpression of ubiquitin-specific protease 22 predicts poor survival in patients with early-stage non-small cell lung cancer. Eur J Histochem.

[27] Yang DD, Cui BB, Sun LY, Zheng HQ, Huang Q, Tong JX, Zhang QF (2011). The co-expression of USP22 and BMI-1 may promote cancer progression and predict therapy failure in gastric carcinoma. Cell Biochem Biophys.

[28] Kosinsky RL, Wegwitz F, Hellbach N, Dobbelstein M, Mansouri A, Vogel T, Begus-Nahrmann Y, Johnsen SA (2015). Usp22 deficiency impairs intestinal epithelial lineage specification in vivo. Oncotarget.

[29] Atanassov BS, Mohan RD, Lan X, Kuang X, Lu Y, Lin K, McIvor E, Li W, Zhang Y, Florens L (2016). ATXN7L3 and ENY2 coordinate activity of multiple H2B Deubiquitinases important for cellular proliferation and tumor growth. Mol Cell.

[30] Bonnet J, Wang CY, Baptista T, Vincent SD, Hsiao WC, Stierle M, Kao CF, Tora L, Devys D (2014). The SAGA coactivator complex acts on the whole transcribed genome and is required for RNA polymerase II transcription. Genes Dev.

[31] Kim J, Guermah M, McGinty RK, Lee JS, Tang Z, Milne TA, Shilatifard A, Muir TW, Roeder RG (2009). RAD6-mediated transcription-coupled H2B ubiquitylation directly stimulates H3K4 methylation in human cells. Cell.

[32] Ng HH, Xu RM, Zhang Y, Struhl K (2002). Ubiquitination of histone H2B by Rad6 is required for efficient Dot1-mediated methylation of histone H3 lysine 79. J Biol Chem.

[33] Sun ZW, Allis CD (2002). Ubiquitination of histone H2B regulates H3 methylation and gene silencing in yeast. Nature.

[34] Hahn MA, Dickson KA, Jackson S, Clarkson A, Gill AJ, Marsh DJ (2012). The tumor suppressor CDC73 interacts with the ring finger proteins RNF20 and RNF40 and is required for the maintenance of histone 2B monoubiquitination. Hum Mol Genet.

[35] Shema E, Tirosh I, Aylon Y, Huang J, Ye C, Moskovits N, Raver-Shapira N, Minsky N, Pirngruber J, Tarcic G (2008). The histone H2B-specific ubiquitin ligase RNF20/hBRE1 acts as a putative tumor suppressor through selective regulation of gene expression. Genes Dev.

[36] Lin Z, Tan C, Qiu Q, Kong S, Yang H, Zhao F, Liu Z, Li J, Kong Q, Gao B, et al. Ubiquitin-specific protease 22 is a deubiquitinase of CCNB1. Cell Discov. 2015;1. 10.1038/celldisc.2015.28 PMC4809424 27030811

[37] Gennaro VJ, Stanek TJ, Peck AR, Sun Y, Wang F, Qie S, Knudsen KE, Rui H, Butt T, Diehl JA (2018). Control of CCND1 ubiquitylation by the catalytic SAGA subunit USP22 is essential for cell cycle progression through G1 in cancer cells. Proc Natl Acad Sci U S A.

[38] Hanahan D, Weinberg RA (2011). Hallmarks of cancer: the next generation. Cell.

[39] Bielenberg DR, Zetter BR (2015). The contribution of angiogenesis to the process of metastasis. Cancer J.

[40] Bikfalvi A (2006). Angiogenesis: health and disease. Ann Oncol.

[41] Macchiarini P, Fontanini G, Hardin MJ, Squartini F, Angeletti CA (1992). Relation of neovascularisation to metastasis of non-small-cell lung cancer. Lancet.

[42] Matsuyama K, Chiba Y, Sasaki M, Tanaka H, Muraoka R, Tanigawa N (1998). Tumor angiogenesis as a prognostic marker in operable non-small cell lung cancer. Ann Thorac Surg.

[43] Koutelou Evangelia, Wang Li, Schibler Andria C., Chao Hsueh-Ping, Kuang Xianghong, Lin Kevin, Lu Yue, Shen Jianjun, Jeter Collene R., Salinger Andrew, Wilson Marenda, Chen Yi Chun, Atanassov Boyko S., Tang Dean G., Dent Sharon Y. R. (2019). USP22 controls multiple signaling pathways that are essential for vasculature formation in the mouse placenta. Development.

[44] Thiery JP (2002). Epithelial-mesenchymal transitions in tumour progression. Nat Rev Cancer.

[45] Moyal L, Lerenthal Y, Gana-Weisz M, Mass G, So S, Wang SY, Eppink B, Chung YM, Shalev G, Shema E (2011). Requirement of ATM-dependent monoubiquitylation of histone H2B for timely repair of DNA double-strand breaks. Mol Cell.

[46] Giannattasio M, Lazzaro F, Plevani P, Muzi-Falconi M (2005). The DNA damage checkpoint response requires histone H2B ubiquitination by Rad6-Bre1 and H3 methylation by Dot1. J Biol Chem.

[47] Lord CJ, Tutt AN, Ashworth A (2015). Synthetic lethality and cancer therapy: lessons learned from the development of PARP inhibitors. Annu Rev Med.

[48] Pinon JD, Labi V, Egle A, Villunger A (2008). Bim and Bmf in tissue homeostasis and malignant disease. Oncogene.

[49] Kepp O, Gottschalk K, Churin Y, Rajalingam K, Brinkmann V, Machuy N, Kroemer G, Rudel T (2009). Bim and Bmf synergize to induce apoptosis in Neisseria gonorrhoeae infection. PLoS Pathog.

[50] Zhang J, Luo N, Tian Y, Li J, Yang X, Yin H, Xiao C, Sheng J, Li Y, Tang B (2017). USP22 knockdown enhanced chemosensitivity of hepatocellular carcinoma cells to 5-Fu by up-regulation of Smad4 and suppression of Akt. Oncotarget.

