# Supplementary material for: Ubiquitin-specific protease 22 is critical to in vivo angiogenesis, growth and metastasis of non-small cell lung cancer
Source: Cell Commun Signal. 2019 Dec 16;17:167. doi: 10.1186/s12964-019-0480-x (PMC6916027; doi:10.1186/s12964-019-0480-x)
Supplement: Supplementary file 1 — Additional file 1 Fig. S1. Differentially expressed gene in USP22-Ko A549 and H1299 cells. Fig. S2. Impact of knockdown of USP22 on H2Bub1/methylatiom of H3K4/K79, cancer cell proliferation, and cell cycle progression. Fig. S3. Negative USP22 IHC stains in adjacent normal cells and tissues. Figure 4. Impact of knockout of USP22 on in vitro migration and invasion of A549 and H1299 cells. Fig. S5. Elevated USP22 in cancer cells survived cisplatin treatment. Fig. S6. Dynamics of P53 in A549 and USP22-Ko cells after 5 and 10 Gy irradiation. [file 12964_2019_480_MOESM1_ESM.pdf]

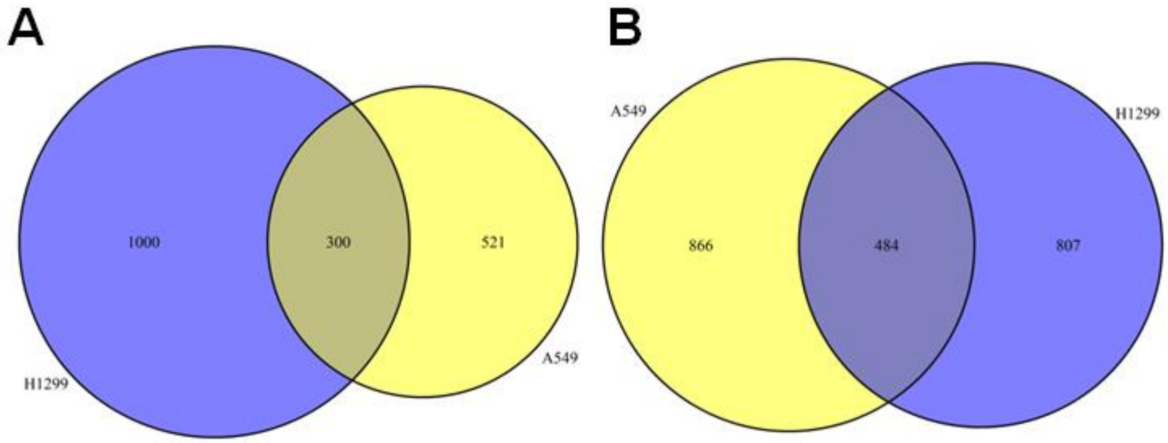

Figure S1. Differentially expressed gene in USP22-Ko A549 and H1299 cells. A. The up-regulated genes. **B.** The downregulated genes in two cells**.** Differentially expressed genes (Log2 FC ≥ 1, *P* < 0.01, FDR < 0.05) in a cell are for these genes that are unanimously modulated in two USP22-/- clones.

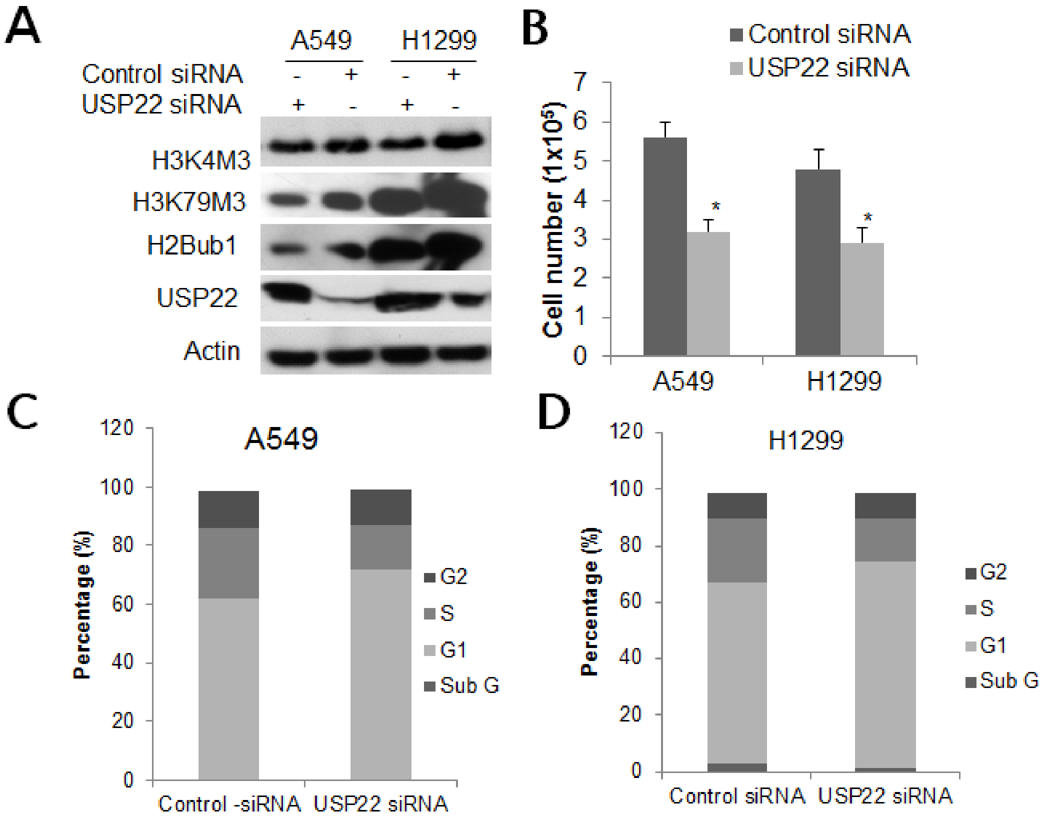

Figure S2. Impact of knockdown of USP22 on H2Bub1/methylatiom of H3K4/K79, cancer cell proliferation, and cell cycle progression. A. Western blot analysis of cell lysates of

A549 and H1299 cells transfected with either USP22 siRNA or scramble (control) siRNA at 72 h post transfection. **B**. Histogram of 72h proliferation (*$P < 0.05$, compared to control siRNA). Transient knockdown of USP22 induced cell cycle arrest in **C**. A549, and **D**. H460 cells. The histogram of cell cycle analysis shows the cell percentage in each phase of these cells transfected with control siRNA or USP22 siRNA.

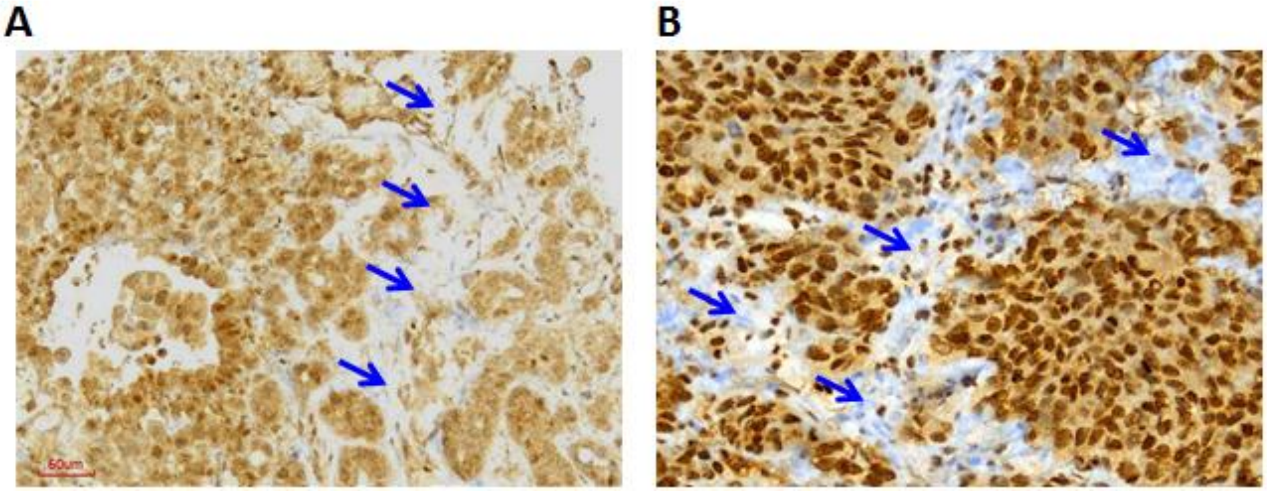

Figure S3. Negative USP22 IHC stains in adjacent normal cells and tissues. A. IHC staining of A549-USP22+/+ xenograft show specific nuclear staining of USP22 is present in cancer cells not in adjacent normal cells. **B.** IHC staining of H1299-USP22+/+ xenograft shows specific nuclear staining of USP22 is present in cancer cells not in normal fibroblast and lymphocyte cells (arrowed, magnification x200).

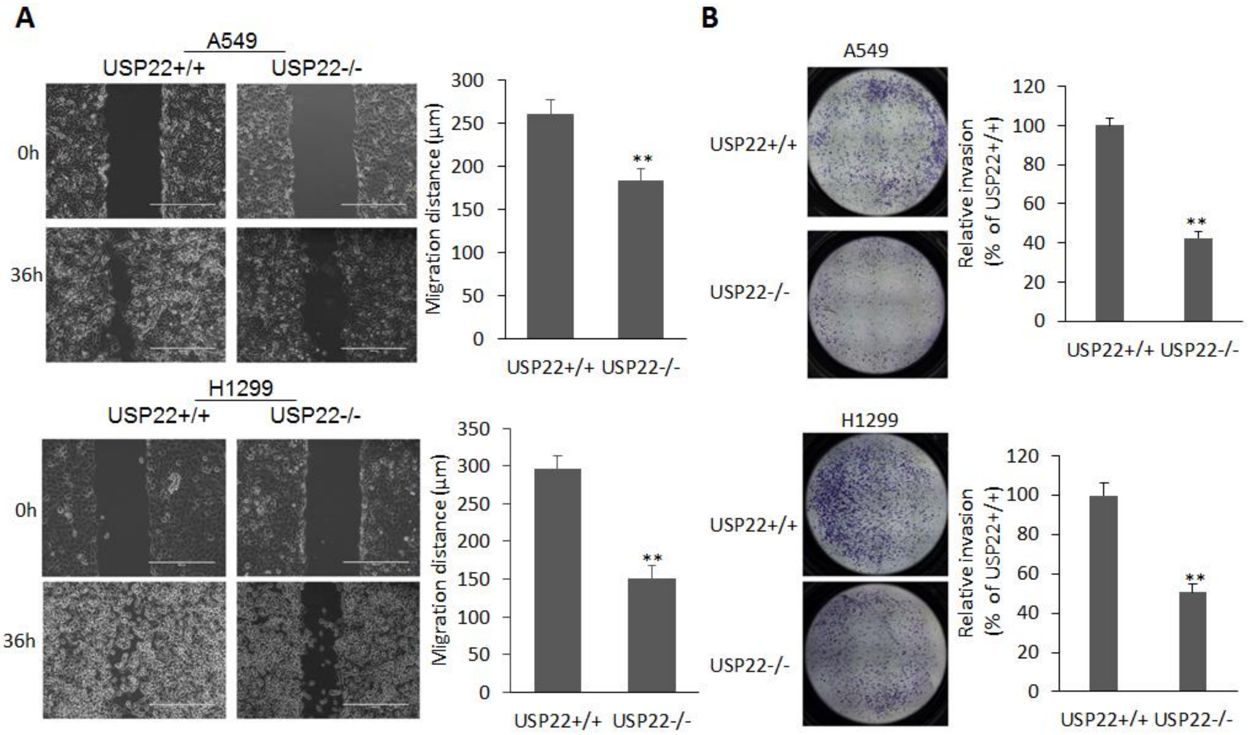

Figure 4: Impact of knockout of USP22 on *in vitro* migration and invasion of A549 and H1299 cells. **A**. Representative photomicrographs of the wound healing assay at 0h and 36h (left panel) and the quantitative analysis of migrating distance in the parental USP22+/+ and USP22-/- cells. **B**. Lower surfaces of Matrigel transwell of USP22/++ and USP22-/- cancer cells (left panel) and the quantitative analysis of invaded cells after 24h culture (right panel). The invaded cell numbers are normalized to that of the parent USP22+/+ cells, and are presented as mean ± SD values of two duplicates per cell type, **P < 0.01, compared with the parent cells.

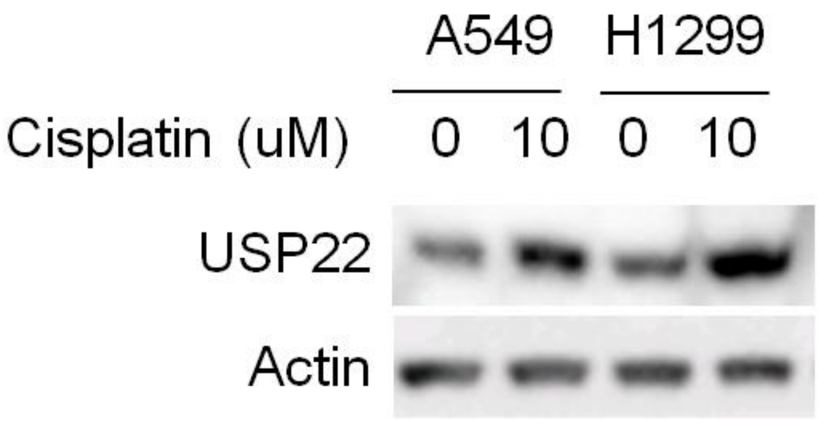

Figure S5. Elevated USP22 in cancer cells survived cisplatin treatment. Cell treated with 10 uM cisplatin for 72h, total protein extract from viable cells were collected for Western blot analysis.

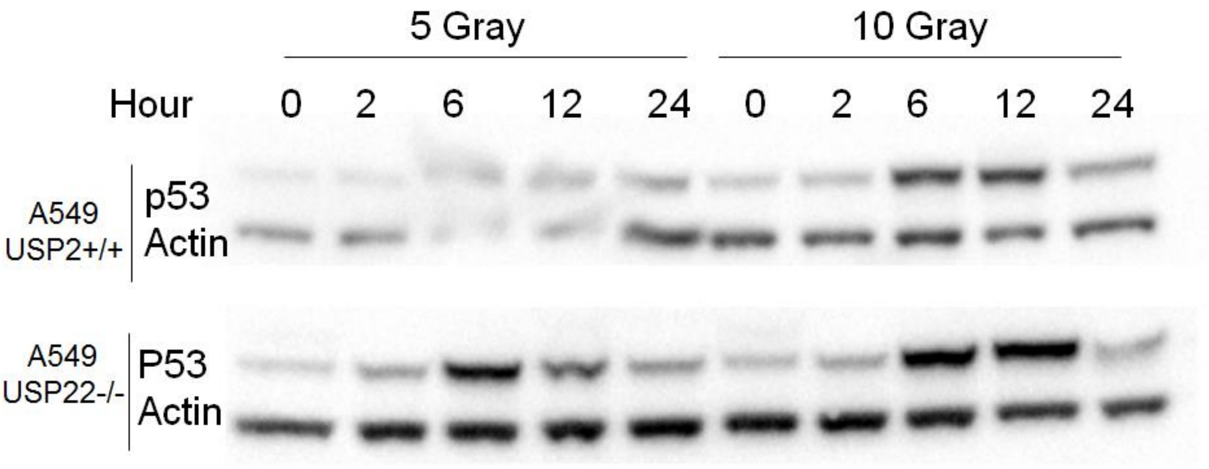

Figure S6. Dynamics of P53 in A549 and USP22-Ko cells after 5 and 10 grays irradiation. Protein was extracted at 2, 6, 12, 24 hours post-irradiation for Western Blot analysis. USP22 for A549-USP22-/- cells.
